# The Therapeutic Effects of Traditional Chinese Medicine for Poor Semen Quality in Infertile Males

**DOI:** 10.3390/jcm7090239

**Published:** 2018-08-24

**Authors:** Shu-Chiu Wang, Shu-Chen Wang, Chia-Jung Li, Ching-Heng Lin, Hsiao-Lin Huang, Liang-Miin Tsai, Chiung-Hung Chang

**Affiliations:** 1 Department of Traditional Chinese Medicine, Tainan Municipal Hospital (Managed By Show Chwan Medical Care Corporation), Tainan 701, Taiwan; wangsc8899@gmail.com; 2 Department of General Laboratory, Tainan Municipal Hospital (Managed By Show Chwan Medical Care Corporation), Tainan 701, Taiwan; 2e0007@tmh.org.tw; 3 Research Assistant Center, Show Chwan Health Memorial Hospital, Changhua 500, Taiwan; nigel6761@gmail.com; 4 Department of Medical Research, Taichung Veterans General Hospital, Taichung 407, Taiwan; epid@vghtc.gov.tw; 5 Department of Public Health, College of Medicine, Fu Jen Catholic University, New Taipei City 242, Taiwan; 6 Department of Health Care Management, National Taipei University of Nursing and Health Sciences, Taipei 112, Taiwan; 7 Institute of Industrial Safety and Disaster Prevention, Chia Nan University of Pharmacy and Science, Tainan 717, Taiwan; hlhuang@mail.cnu.edu.tw; 8 Department of Internal Medicine, National Cheng Kung University Hospital, College of Medicine, National Cheng Kung University, Tainan 704, Taiwan; 9 Department of Internal Medicine, Tainan Municipal Hospital (Managed By Show Chwan Medical Care Corporation), Tainan 701, Taiwan; 10 Department of Traditional Chinese Medicine, Taichung Veterans General Hospital, Taichung 407, Taiwan

**Keywords:** male infertility, semen parameters, traditional Chinese medicine

## Abstract

Poor sperm quality is one of the main factors of male infertility. Traditional Chinese Medicine (TCM) has been used frequently in clinical practice in many countries to treat a wide array of infertile problems. To further understand the effects of TCM on semen quality, we retrospectively enrolled patients with male infertility and poor semen quality at the Tainan Municipal Hospital in Taiwan between 2013 and 2016. Semen quality analysis in accordance with the WHO criteria is an essential step in the evaluation of male fertility status. Associations between the semen parameters and body mass index, smoking status, alcohol use, duration of infertility, and age were also analyzed. A total of 126 male infertility patients with abnormal semen analysis were included in this study: 50 TCM users and 13 TCM non-users. The basic characteristics of the two groups were not significantly different. TCM users account for 92.5% of the total semen improvement subjects. In conclusion, TCM supplementation may have a beneficial role as improving sperm quality for infertility patients.

## 1. Introduction

Infertility is an important global reproductive health issue. According to the International Committee for Monitoring Assisted Reproductive Technology (ICMART) and the World Health Organization (WHO), the clinical definition of infertility is “a disease of the reproductive system defined by the failure to achieve a clinical pregnancy after 12 months or more of regular unprotected sexual intercourse” [1]. In 2010, the WHO estimates that approximately 48.5 million couples (8–12% of couples) worldwide have infertility [2]. The prevalence of infertility differs between countries and regions.

The distribution of infertility due to male factors is between 20% and 70% [3]. The main causes of male infertility are genetic factors, hormone disorders (such as hypothyroidism, hyperprolactinemia), physical problems (such as varicocele, damaged sperm ducts), lifestyle problems (such as smoking, alcohol, or drug abuse), previous infections, environmental causes (such as plasticizer, radiation, or X-rays), and unexplained male infertility [4,5,6,7,8,9,10]. Owing to marriages in later life, lifestyle changes, stress in life and work, and environmental pollution, the quality of human sperm has declined significantly [11]. Poor semen quality is a major factor of male infertility. The pathological processes that lead to unexplained abnormalities in sperm parameters are often poorly understood [12].

Nevertheless, various drugs or supplements have been shown to improve poor-quality sperm in men with infertility. However, some studies have revealed contrasting results. Antioxidant vitamins or minerals, such as vitamins E, selenium, zinc, and L-carnitine, have positively affected semen quality [11,13]. However, some drugs, such as androgens and human chorionic gonadotropin (hCG)/human menopausal gonadotropin (hMG), are not effective for the treatment of low semen quality [14,15]. Thus, approximately 30–40% of men with infertility seek one or more alternative therapies such as herbal medicines or acupuncture [16,17]. In animal studies, the treatment with herbal medicine has been shown to significantly improve male infertility [18,19,20,21]. A number of studies have revealed the active components of Traditional Chinese Medicine (TCM) that bolster blood circulation in the reproductive system and that control testosterone secretion. They also have an important role in the enhancement of the parameters of healthy sperm, including sperm count, viability, motility, and morphology. Herbal medicines have also shown positive effects in men with very poor sperm quality [11,22,23]. Given the insufficient quantity of literature and small number of studies, it is unsurprising that a systematic review has shown that the evidence on whether herbal medicines affect sperm quantity of male fertility is insufficient to draw firm conclusions.

The present study was designed to evaluate the effects of TCM used for the treatment of male infertility on the parameters of semen with respect to the WHO reference values. In a recent retrospective cohort study, we reviewed data collected from men with infertility that were treated at Tainan Municipal Hospital (managed by Show Chwan Medical Care Corporation, Tainan, Taiwan) for semen analysis between 2013 and 2016.

## 2. Materials and Methods

### 2.1. Data Source and Study Design

The patient data in our study were retrieved from the outpatient records of Tainan Municipal Hospital (managed by Show Chwan Medical Care Corporation, Tainan, Taiwan) in southern Taiwan, between January 2013 and December 2016. We enrolled patients diagnosed with male infertility by using the ICD-9-CM (the International Classification of Diseases, 9th Revision, Clinical Modification) code 6069. The collected data contained age, duration of infertility, BMI (body mass index), diagnosis, and semen characteristics, including sperm count, morphology, and motility.

The eligibility criteria for inclusion were: duration of infertility ≥1 year; men with infertility with poor semen quality (up to three months, considered baseline); a follow-up semen analysis was performed after three months. The definition of “poor semen quality” was abnormal semen parameters confirmed in accordance with the 2010 WHO criteria within three months of visiting our hospital, including oligozoospermia, teratozoospermia, and asthenozoospermia, or a combination of these three parameters. Azoospermia, aspermia, varicocele, recent urogenital infections, Y chromosome deletions, abnormal karyotypes, endocrinopathies, and hormonal disorders were excluded from the study. If the patient was lost to follow-up or had incomplete semen analysis, they were to be excluded. Subjects that patients that received TCM were assigned as TCM users; other patients were assigned to the group of TCM nonusers. An individual formula was prescribed for the patient’s condition, based on the theories of TCM. 

The determination of the patient’s condition involved an analysis of the clinical data of symptoms, physical signs, abnormal semen parameters, and lifestyle factors by Chinese medicine physicians with at least five years of experience in infertility treatment. Participants who received other medicines, certain micronutrients, and antioxidant supplementation during TCM treatment were also excluded. The semen analysis data were compared between the TCM users and the TCM nonusers.

### 2.2. Semen Analysis

Semen samples were collected by masturbation under hygienic conditions after an abstinence period of 2–7 days, liquefied at 37 °C, and then analyzed by two experienced technicians in accordance with the standard protocols described in the WHO laboratory manual [24]. The sperm parameters include sperm concentration (million/mL), total (progressive + non-progressive) motility (expressed as a percentage), and morphologically normal forms (expressed as a percentage). All parameters were defined and interpreted in accordance with WHO guidelines [25].

### 2.3. Statistical Analysis

To compare the demographic characteristics between TCM users and nonusers, mean and standard deviation (SD) values were calculated for age, duration of infertility, BMI, number and percentage of smokers, alcohol use, and infertility type. The *t*-test was used to test the difference in the means of continuous variables and the Chi-squared test was used to evaluate the distribution difference for category variables. To compare the semen improvement between TCM users and nonusers, the odds ratios (ORs) and corresponding 95% confidence intervals (CIs) were estimated by single variable and multivariable logistic regression models. The difference in semen parameters between before and after intervention was considered as a continuous outcome, and a linear regression analysis was performed to find the beta value and corresponding standard error (SE) values for TCM users and TCM nonusers. All data management and analyses were performed by using SAS 9.4 software (SAS Institute, Cary, NC, USA). In two-sided tests, *p*-values of less than 0.05 indicated statistical significance.

### 2.4. Ethics

As this was a retrospective study, informed consent was not available. This study was approved by the Institutional Review Board (IRB) of the Show Chwan Medical Foundation (IRB No. 1070202). The organized IRB operates in accordance with Good Clinical Practice, and all applicable laws and regulations.

## 3. Results

**Figure 1:**
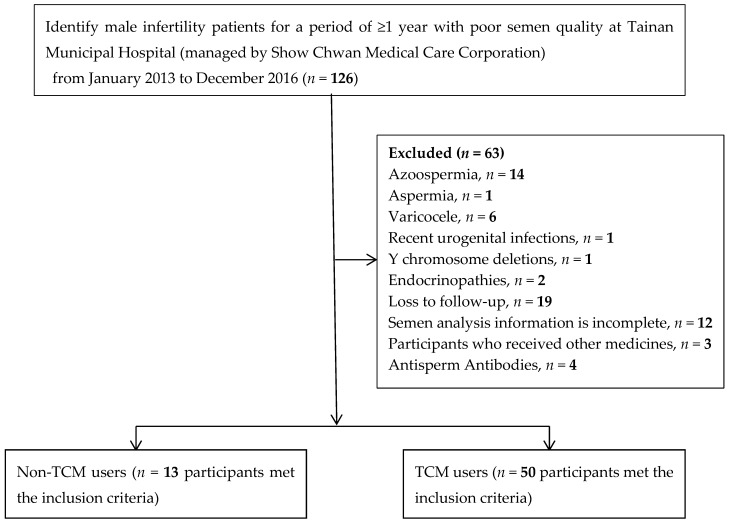
Flow diagram for the selection of eligible studies and subjects.

In this retrospective study, we identified 126 men with infertility with abnormal semen analysis, according to the 2010 WHO guidelines, at Tainan Municipal Hospital (managed by Show Chwan Medical Care Corporation, Tainan, Taiwan) between January 2013 and December 2016 (Figure 1). Sixty-three men were excluded from the initial analysis owing to: Azoospermia (*n* = 14), aspermia (*n* = 1), varicocele (*n* = 6), recent urogenital infections (*n* = 1), Y chromosome deletions (*n* = 1), endocrinopathies (*n* = 2), loss to follow-up (*n* = 19), incomplete semen analysis information (*n* = 12), other medicines received (*n* = 3), and antisperm antibodies (*n* = 4) (Figure 1). In total, 13 patients did not receive TCM treatment and 50 patients received TCM treatment.

The demographic feature of TCM users and TCM nonusers are shown in Table 1. In total, this study comprised 50 TCM users and 13 TCM nonusers. The baseline descriptive characteristics of both groups were similar. The mean age of TCM users and TCM nonusers was 37.7 and 37.6 years of age, respectively, which was not significantly different (*p* = 0.97). The mean duration of infertility was nearly three years for study subjects and was predominantly primary infertility.

The results of the semen improvement are shown in Table 2. In the semen improvement subjects, 92.5% of patients were TCM users. After adjustment for age, duration of infertility, BMI, history of smoking and alcohol use, and infertility type, the TCM users had a 10.7-fold better semen improvement than the TCM nonusers (OR = 10.7, 95% CI = 2.28–50.4).

**Figure 2:**
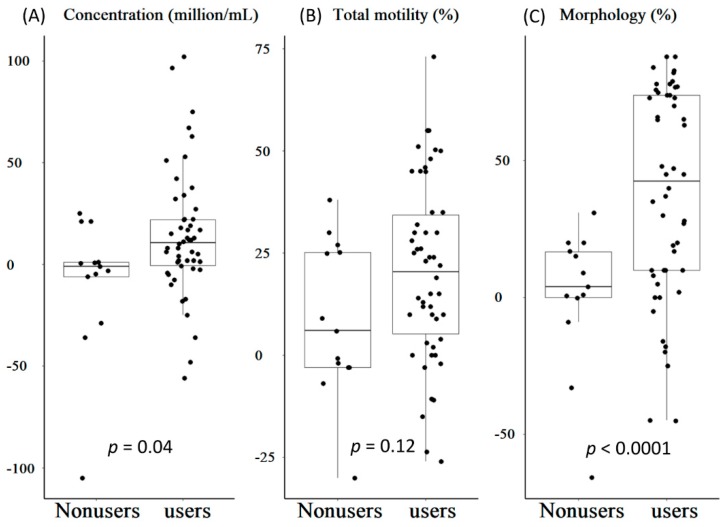
The changed value of semen parameter and linear regression analysis for changed value between TCM users and nonusers. (**A**) Concentration; (**B**) Total motility; (**C**) Morphology.

The changes in semen parameters and linear regression analysis of the changes in values between TCM users and TCM nonusers were determined. The mean changes in concentration, total motility, and morphology in TCM users were 14.3 million/mL, 20.4%, and 38.1%, respectively, and −8.86 million/mL, 8.79%, and 0.71% in TCM nonusers. Except for total motility change, the TCM users exhibited significantly better concentration and morphology than TCM nonusers (Figure 2).

## 4. Discussion

### 4.1. Summary of Evidence

Poor sperm quality is known to be an important factor in male fertility [25]. Infertility is considered to arise from male factors when the sperm parameters (sperm concentration, motility, and/or morphology) of men were below the WHO normal values. The main purpose of this study was to investigate whether TCM was effective for the improvement of sperm quality. In this study, we retrospectively analyzed the medical records of male subjects and for inclusion criteria between January 2013 and December 2016 at Tainan Municipal Hospital in southern Taiwan. During the study period, a total of 50 eligible men with infertility receiving TCM treatment were enrolled and considered to be the TCM users. The TCM nonusers comprised 13 men with infertility who had once received any medicine or micronutrient supplementation for the improvement of their semen quality or untreated. The baseline characteristics of the two groups were not significantly different (Table 1).

In this study, it was found that TCM users account for 92.5% of the total semen improvement subjects, the other 7.5% is up to TCM nonusers (Table 2). ”Semen improvement” was considered to represent an improvement in sperm parameters in the presence or absence of TCM treatment, and sperm concentration or motility or morphology complied with the WHO guidelines in two sperm analyses, collected three months apart. The TCM users had 10.7-fold better semen improvement than TCM nonusers after adjustment for age, duration of infertility, BMI, history of smoking and alcohol use, and infertility type (Table 2). Our data were in agreement with that of the previous studies that clearly demonstrated that TCM exerted a positive effect on all sperm parameters [26,27]. 

### 4.2. Mechanism of Traditional Chinese Herbs for the Treatment of Poor Semen Quality in Male Infertility

The physiological mechanisms for most TCMs used in male infertility are unclear. Currently, pharmacological studies into the mechanism of action of a variety of TCMs have been performed. The TCM components of *Cuscuta chinensis* Lam., *Epimedium brevicornum* Maxim., *Panax ginseng*, *Morinda officinalis*, *Lycium chinense*, and ginger have been explored. Numerous studies have reported that these ingredients reduce reactive oxygen species (ROS), increase sperm motility, and effectively increase conception rates. A number of studies have shown that that sperm cells are particularly susceptible to the damage induced by excessive ROS generation, which decreases the sperm function and results in male infertility [28,29,30]. Approximately 25% to 40% of infertile men were reported to have high levels of ROS detected in their semen [29,31,32]. Antioxidant protection is a vital element in the maintenance of sperm membrane integrity and the prevention of lipid peroxidation of the sperm plasma membrane, which may lead to sperm dysfunction and cell death [33]. Therefore, antioxidants may protect against ROS-induced male infertility and enhance their ability to contribute to successful conception. Vitamin C, vitamin E, selenium, and CoQ10 are well known antioxidants for the improvement of semen parameters in infertile men [34]. In addition, recent studies have shown that *Cuscuta chinensis* Lam. and *Lycium chinense* act as antioxidants in maintaining sperm quality [35,36]. Wu-Zi-Yan-Zong-Wan, which contains *Cuscuta chinensis* Lam. and *Lycium chinense*, has a protective effect against oxidative damage of mitochondrial DNA (mtDNA) in aged men [37,38]. Moreover, studies in animals showed that Wu-Zi-Yan-Zong-Wan exhibited protective effects against ionizing radiation-induced testicular damage, which may have been related to its antioxidant abilities [39]. Zhao et al. demonstrated that Wu-Zi-Yan-Zong-Wan might ameliorate sperm quality by boosting semen parameters and decreasing DNA damage in patients with oligoasthenozoospermia [26]. Omirinde et al. demonstrated that *Cuscuta australis* enhanced sperm morphology, decreased defects of the head and mid-piece of spermatozoa, and invigorated the reproductive system through its strong antioxidant properties [40].

The decrease of sperm counts and testosterone levels in men in recent decades is an indication that a low sperm count may reduce fertility [41]. Testosterone reduction results in lower energy levels, weaker erections, lower libido, and reduced sperm count. Exogenous testosterone supplementation suppresses the hypothalamic-pituitary-gonadal axis, resulting in a decrease in overall testosterone levels and sperm production in the testes [42]. However, as noted above, TCM therapy represents an alternative treatment; instead of replacing testosterone, the body is encouraged to increase natural testosterone production. The results of the present study indicated that *Panax ginseng* may increase the production of testosterone and the spermatid populations through the suppression of ROS production [19]. In addition, ginsenosides, the active components of ginseng, were shown to increase sperm concentrations, motility, morphology, and viability [22,43] and facilitate erectile dysfunction in males [44]. Ba Ji Tian (*Morinda officinalis*) increased the production of testosterone, reduced the concentration of cortisol, improved the quality of the sperm, and protected the DNA of human sperm from H_2_O_2_ damage [45].

Human seminal plasma contains several trace elements, especially Zn, that perform important physiological functions in semen. Many studies demonstrated that Zn plays an important role in sperm quality, and a reduction in Zn concentration will yield low quality sperm, which reduces the probability of fertilization [46]. Zhang et al. demonstrated that *Lycium barbarum* polysaccharides (LBP-4) were composed of six types monosaccharides that could enhance the food conversion rate and the content of Zn and Fe in mice [47]. Many trace elements were detected in Zn, Fe, and Cu in *Morinda officinalis* How, and polysaccharides extracted from *M. officinalis* How improved many trace elements, including Zn, Mg, and Ca, in ovariectomized rats [48].

The main factor determining factor of sperm count is the number of Sertoli cells in the testes. Sertoli cells play a key role during the development of germ cells into sperm. Each Sertoli cell can only support a certain number of germ cells; consequently, the number of Sertoli cells per testis determines the total amount of sperm production [49]. An in vitro study showed that icariin (ICA), a major constituent flavonoid from the Chinese medical herb *Epimedium brevicornum* Maxim., promoted the proliferation of Sertoli cells in vitro through the activation of the ERK1/2 signaling pathway, which was thought to increase the overall level of sperm production and male reproductive ability [50]. TCM also possessed an immunoregulatory effect [51], anti-inflammatory effect, [52] and ameliorated infection [53]; however, these were not discussed in our study, because these participants were excluded from our analysis.

### 4.3. Changes in Semen Parameters Values

All semen parameters were significantly improved after three months of treatment with TCM. Except for the sperm motility, the concentration of TCM users was significantly improved compared to non-users of TCM, especially with a highly significant improvement in morphology. In TCM nonusers, a net increase was observed for motility change only (Figure 2), but this change did not meet the WHO criterion. This was only related to the health improvements in TCM nonusers through lifestyle modifications such as nutrition quality improvement, regular exercise, avoiding high temperatures, minimizing exposure to plasticizers, and reducing cigarette consumption.

### 4.4. Adverse Effects

In the study, no adverse reactions were noted in the clinical medical records of either treatment group.

## 5. Conclusions

The majority of these studies reported a positive relationship between poor semen quality and male infertility, resulting in a low birth rate and an aging population. Thus, the solution of this problem by using TCM is particularly interesting. This retrospective study demonstrated that TCM significantly improved sperm quality. The present study indicated that TCM can be used as an alternative treatment for male infertility with poor sperm quality. Our data also support the possible use of the TCM as a therapeutic strategy that can improve sperm quality in infertility patients. However, more randomized clinical trials and further research are required to reinforce our findings.

## Figures and Tables

**Table 1:** Baseline characteristic between Traditional Chinese Medicine (TCM) users and nonusers.

Variable	TCM Nonusers	TCM Users	*p*-Value
	*N* = 13 (%)	*N* = 50 (%)	
Age, years (SD) *	37.6 (3.75)	37.7 (6.31)	0.97
Duration of infertility, year (SD) *	2.77 (1.30)	4.43 (3.46)	0.009
Body mass index, mean (SD) *	25.7 (4.42)	25.5 (3.39)	0.84
Smokers	2 (15.4)	7 (14.0)	0.90
Alcohol use	0 (0)	3 (6.0)	0.37
Infertility type			0.46
Primary	13 (100)	48 (96.0)	
Secondary	0 (0)	2 (4.00)	

* Statistics: *t*-test.

**Table 2:** Odds ratios and 95% confidence intervals for semen improvement.

Variable	Semen Improvement		Crude OR (95% CI)	Adjusted OR (95% CI)
	No *N* = 23 (%)	Yes *N* = 40 (%)		
TCM				
No	10 (43.5)	3 (7.50)	ref	ref
Yes	13 (56.5)	37 (92.5)	9.49 (2.26–39.9)	10.7 (2.28–50.4)
Age group, years				
<40	14 (60.9)	32 (80.0)	ref	ref
≥40	9 (39.1)	8 (20.0)	0.39 (0.12–1.22)	0.37 (0.10–1.40)
Duration of infertility, yeas				
<3	9 (39.1)	16 (40.0)	ref	ref
≥3	14 (60.9)	24 (60.0)	0.96 (0.34–2.75)	1.04 (0.27–4.02)
BMI				
<24	7 (30.4)	14 (35.0)	ref	ref
≥24	16 (69.6)	26 (65.0)	0.81 (0.27–2.44)	0.84 (0.22–3.16)
Smokers (ref: non-smokers)	5 (21.7)	4 (10.0)	0.40 (0.10–1.67)	0.27 (0.04–1.72)
Alcohol use (ref: nonusers)	1 (4.35)	2 (5.00)	1.16 (0.10–13.5)	1.44 (0.09–22.1)
Infertility type				
Primary	22 (95.7)	39 (97.5)	ref	ref
Secondary	1 (4.35)	1 (2.50)	0.56 (0.03–9.47)	0.21 (0.01–4.30)

Semen non-improvement: Post-intervention meets one of the following: 1. Concentration: <15 million/mL; 2. Total motility: <40%; 3. Morphology: <4%.
